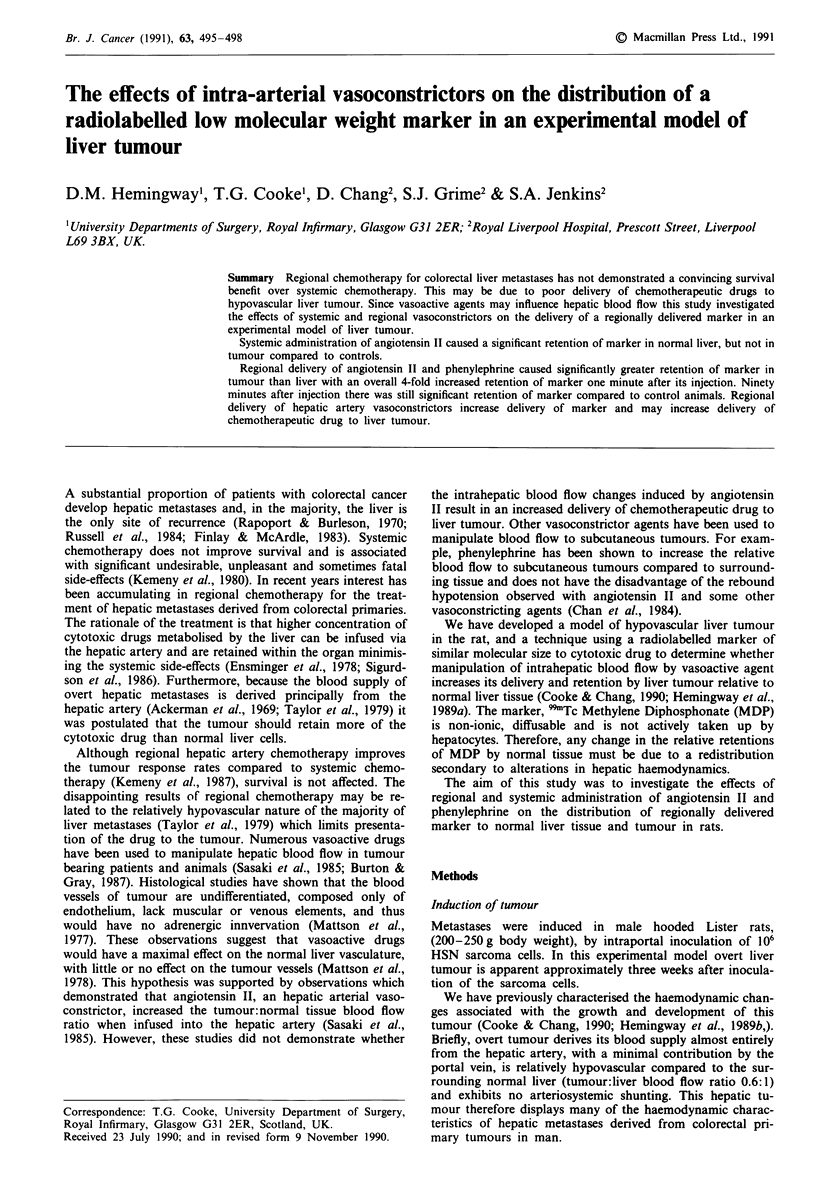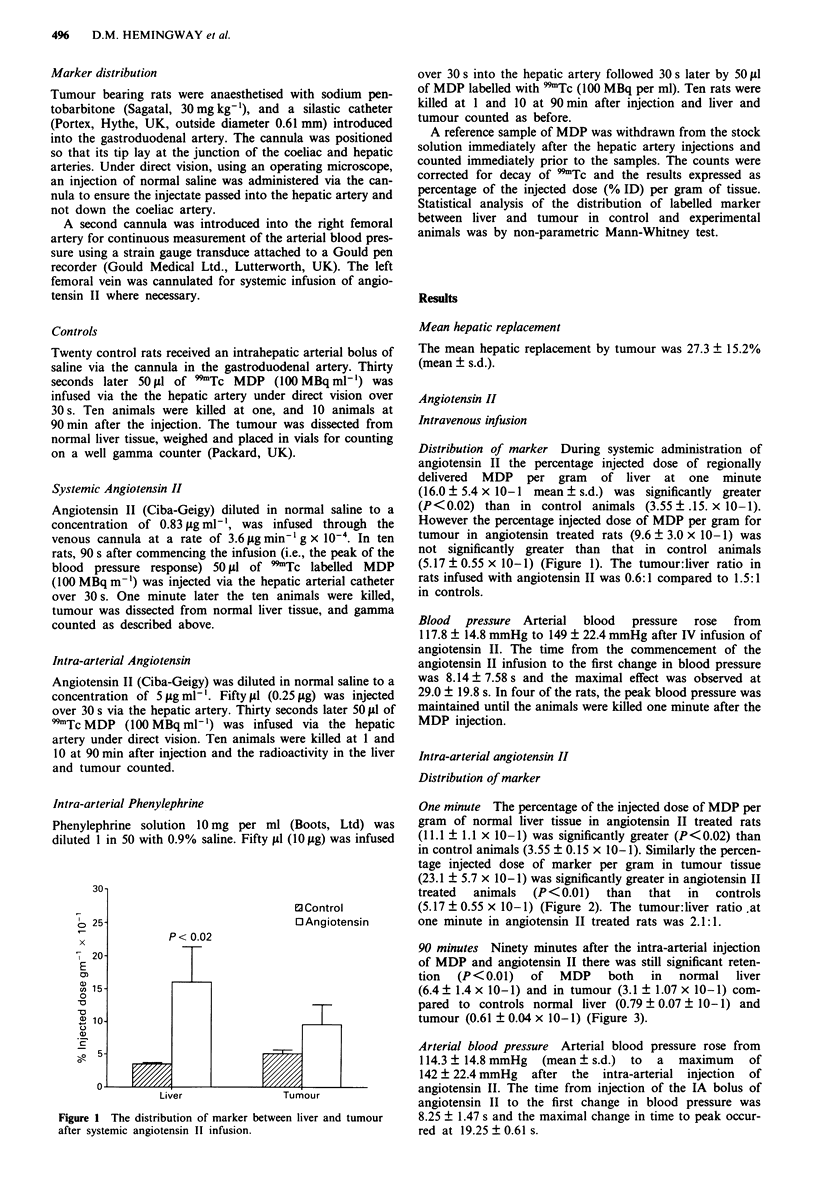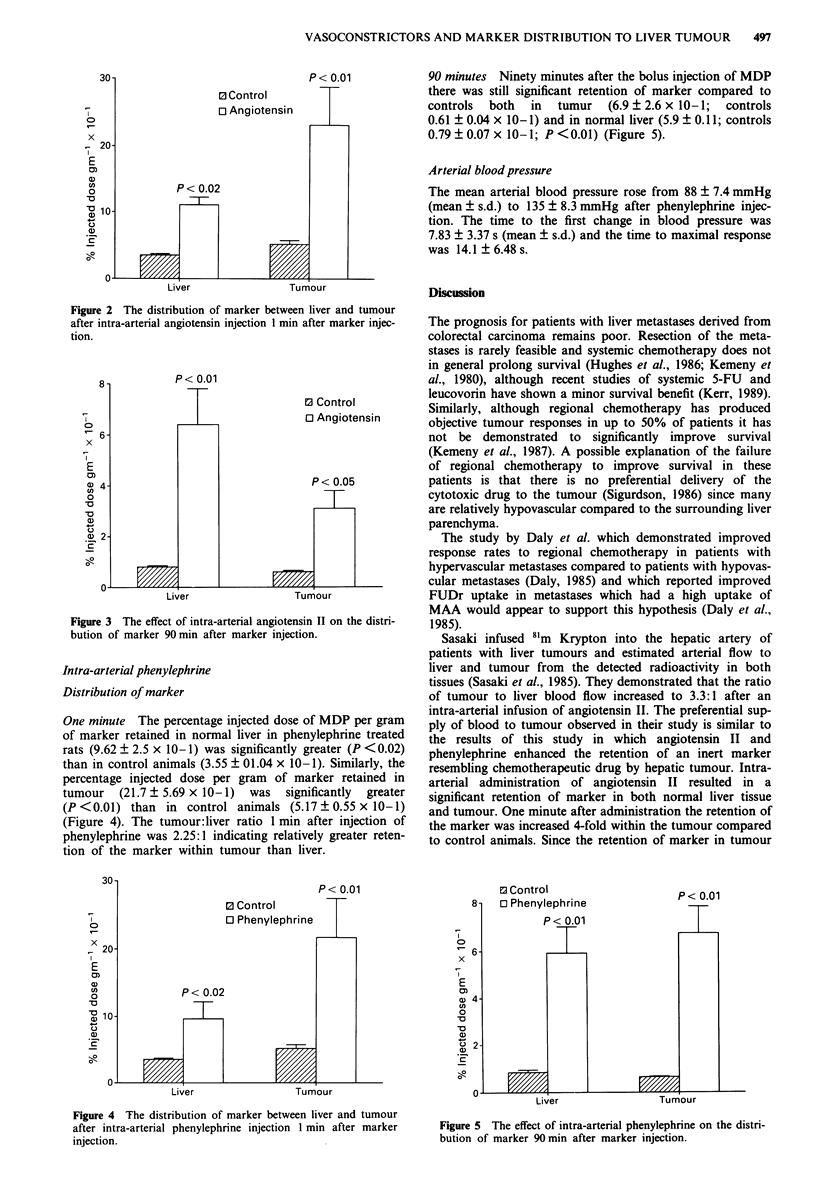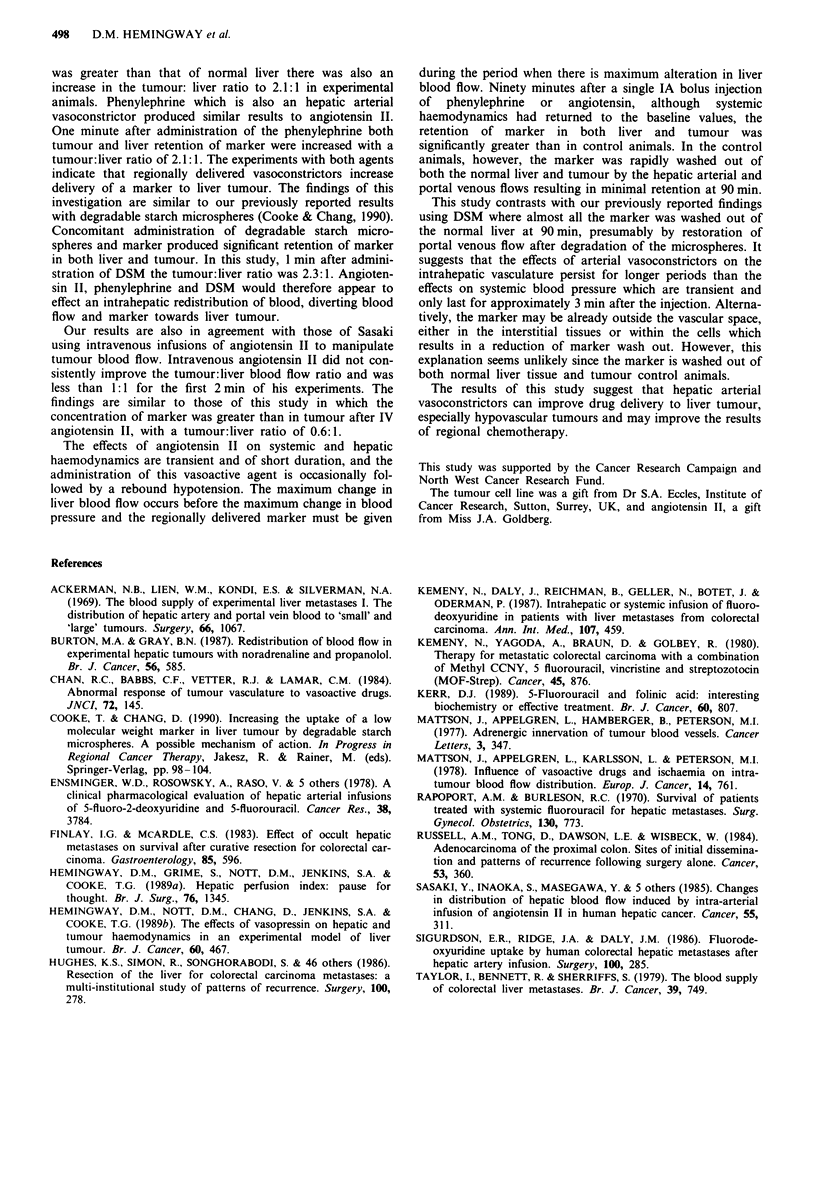# The effects of intra-arterial vasoconstrictors on the distribution of a radiolabelled low molecular weight marker in an experimental model of liver tumour.

**DOI:** 10.1038/bjc.1991.118

**Published:** 1991-04

**Authors:** D. M. Hemingway, T. G. Cooke, D. Chang, S. J. Grime, S. A. Jenkins

**Affiliations:** University Department of Surgery, Royal Infirmary, Glasgow, UK.

## Abstract

Regional chemotherapy for colorectal liver metastases has not demonstrated a convincing survival benefit over systemic chemotherapy. This may be due to poor delivery of chemotherapeutic drugs to hypovascular liver tumour. Since vasoactive agents may influence hepatic blood flow this study investigated the effects of systemic and regional vasoconstrictors on the delivery of a regionally delivered marker in an experimental model of liver tumour. Systemic administration of angiotensin II caused a significant retention of marker in normal liver, but not in tumour compared to controls. Regional delivery of angiotensin II and phenylephrine caused significantly greater retention of marker in tumour than liver with an overall 4-fold increased retention of marker one minute after its injection. Ninety minutes after injection there was still significant retention of marker compared to control animals. Regional delivery of hepatic artery vasoconstrictors increase delivery of marker and may increase delivery of chemotherapeutic drug to liver tumour.


Br. J. Cancer (1991), 63, 495-498 © Macmillan Press Ltd., 1991

### The effects of intra-arterial vasoconstrictors on the distribution of a radiolabelled low molecular weight marker in an experimental model of liver tumour

D.M. Hemingway[1], T.G. Cooke[1], D. Chang[2], S.J. Grime[2] & S.A. Jenkins[2]

[1]*University Departments of Surgery, Royal Infirmary, Glasgow G31 2ER;* [2]*Royal Liverpool Hospital, Prescott Street, Liverpool L69 3BX, UK.*

**Summary** Regional chemotherapy for colorectal liver metastases has not demonstrated a convincing survival benefit over systemic chemotherapy. This may be due to poor delivery of chemotherapeutic drugs to hypovascular liver tumour. Since vasoactive agents may influence hepatic blood flow this study investigated the effects of systemic and regional vasoconstrictors on the delivery of a regionally delivered marker in an experimental model of liver tumour.

Systemic administration of angiotensin II caused a significant retention of marker in normal liver, but not in tumour compared to controls.

Regional delivery of angiotensin II and phenylephrine caused significantly greater retention of marker in tumour than liver with an overall 4-fold increased retention of marker one minute after its injection. Ninety minutes after injection there was still significant retention of marker compared to control animals. Regional delivery of hepatic artery vasoconstrictors increase delivery of marker and may increase delivery of chemotherapeutic drug to liver tumour.

A substantial proportion of patients with colorectal cancer develop hepatic metastases and, in the majority, the liver is the only site of recurrence (Rapoport & Burleson, 1970; Russell *et al.*, 1984; Finlay & McArdle, 1983). Systemic chemotherapy does not improve survival and is associated with significant undesirable, unpleasant and sometimes fatal side-effects (Kemeny *et al.*, 1980). In recent years interest has been accumulating in regional chemotherapy for the treatment of hepatic metastases derived from colorectal primaries. The rationale of the treatment is that higher concentration of cytotoxic drugs metabolised by the liver can be infused via the hepatic artery and are retained within the organ minimising the systemic side-effects (Ensminger *et al.*, 1978; Sigurdson *et al.*, 1986). Furthermore, because the blood supply of overt hepatic metastases is derived principally from the hepatic artery (Ackerman *et al.*, 1969; Taylor *et al.*, 1979) it was postulated that the tumour should retain more of the cytotoxic drug than normal liver cells.

Although regional hepatic artery chemotherapy improves the tumour response rates compared to systemic chemotherapy (Kemeny *et al.*, 1987), survival is not affected. The disappointing results of regional chemotherapy may be related to the relatively hypovascular nature of the majority of liver metastases (Taylor *et al.*, 1979) which limits presentation of the drug to the tumour. Numerous vasoactive drugs have been used to manipulate hepatic blood flow in tumour bearing patients and animals (Sasaki *et al.*, 1985; Burton & Gray, 1987). Histological studies have shown that the blood vessels of tumour are undifferentiated, composed only of endothelium, lack muscular or venous elements, and thus would have no adrenergic innvervation (Mattson *et al.*, 1977). These observations suggest that vasoactive drugs would have a maximal effect on the normal liver vasculature, with little or no effect on the tumour vessels (Mattson *et al.*, 1978). This hypothesis was supported by observations which demonstrated that angiotensin II, an hepatic arterial vasoconstrictor, increased the tumour:normal tissue blood flow ratio when infused into the hepatic artery (Sasaki *et al.*, 1985). However, these studies did not demonstrate whether the intrahepatic blood flow changes induced by angiotensin II result in an increased delivery of chemotherapeutic drug to liver tumour. Other vasoconstrictor agents have been used to manipulate blood flow to subcutaneous tumours. For example, phenylephrine has been shown to increase the relative blood flow to subcutaneous tumours compared to surrounding tissue and does not have the disadvantage of the rebound hypotension observed with angiotensin II and some other vasoconstricting agents (Chan *et al.*, 1984).

We have developed a model of hypovascular liver tumour in the rat, and a technique using a radiolabelled marker of similar molecular size to cytotoxic drug to determine whether manipulation of intrahepatic blood flow by vasoactive agent increases its delivery and retention by liver tumour relative to normal liver tissue (Cooke & Chang, 1990; Hemingway *et al.*, 1989*a*). The marker, $^{99m}$Tc Methylene Diphosphonate (MDP) is non-ionic, diffusable and is not actively taken up by hepatocytes. Therefore, any change in the relative retentions of MDP by normal tissue must be due to a redistribution secondary to alterations in hepatic haemodynamics.

The aim of this study was to investigate the effects of regional and systemic administration of angiotensin II and phenylephrine on the distribution of regionally delivered marker to normal liver tissue and tumour in rats.

### Methods

### *Induction of tumour*

Metastases were induced in male hooded Lister rats, (200-250 g body weight), by intraportal inoculation of $10^6$ HSN sarcoma cells. In this experimental model overt liver tumour is apparent approximately three weeks after inoculation of the sarcoma cells.

We have previously characterised the haemodynamic changes associated with the growth and development of this tumour (Cooke & Chang, 1990; Hemingway *et al.*, 1989*b*,). Briefly, overt tumour derives its blood supply almost entirely from the hepatic artery, with a minimal contribution by the portal vein, is relatively hypovascular compared to the surrounding normal liver (tumour:liver blood flow ratio 0.6:1) and exhibits no arteriosystemic shunting. This hepatic tumour therefore displays many of the haemodynamic characteristics of hepatic metastases derived from colorectal primary tumours in man.

Correspondence: T.G. Cooke, University Department of Surgery, Royal Infirmary, Glasgow G31 2ER, Scotland, UK.
Received 23 July 1990; and in revised form 9 November 1990.

*Marker distribution*

Tumour bearing rats were anaesthetised with sodium pentobarbitone (Sagatal, 30 mg $kg^{-1}$), and a silastic catheter (Portex, Hythe, UK, outside diameter 0.61 mm) introduced into the gastroduodenal artery. The cannula was positioned so that its tip lay at the junction of the coeliac and hepatic arteries. Under direct vision, using an operating microscope, an injection of normal saline was administered via the cannula to ensure the injectate passed into the hepatic artery and not down the coeliac artery.

A second cannula was introduced into the right femoral artery for continuous measurement of the arterial blood pressure using a strain gauge transduce attached to a Gould pen recorder (Gould Medical Ltd., Lutterworth, UK). The left femoral vein was cannulated for systemic infusion of angiotensin II where necessary.

*Controls*

Twenty control rats received an intrahepatic arterial bolus of saline via the cannula in the gastroduodenal artery. Thirty seconds later 50 µl of $^{99m}Tc$ MDP (100 MBq $ml^{-1}$) was infused via the the hepatic artery under direct vision over 30 s. Ten animals were killed at one, and 10 animals at 90 min after the injection. The tumour was dissected from normal liver tissue, weighed and placed in vials for counting on a well gamma counter (Packard, UK).

*Systemic Angiotensin II*

Angiotensin II (Ciba-Geigy) diluted in normal saline to a concentration of 0.83 µg $ml^{-1}$, was infused through the venous cannula at a rate of 3.6 µg $min^{-1}$ g × $10^{-4}$. In ten rats, 90 s after commencing the infusion (i.e., the peak of the blood pressure response) 50 µl of $^{99m}Tc$ labelled MDP (100 MBq $m^{-1}$) was injected via the hepatic arterial catheter over 30 s. One minute later the ten animals were killed, tumour was dissected from normal liver tissue, and gamma counted as described above.

*Intra-arterial Angiotensin*

Angiotensin II (Ciba-Geigy) was diluted in normal saline to a concentration of 5 µg $ml^{-1}$. Fifty µl (0.25 µg) was injected over 30 s via the hepatic artery. Thirty seconds later 50 µl of $^{99m}Tc$ MDP (100 MBq $ml^{-1}$) was infused via the hepatic artery under direct vision. Ten animals were killed at 1 and 10 at 90 min after injection and the radioactivity in the liver and tumour counted.

*Intra-arterial Phenylephrine*

Phenylephrine solution 10 mg per ml (Boots, Ltd) was diluted 1 in 50 with 0.9% saline. Fifty µl (10 µg) was infused over 30 s into the hepatic artery followed 30 s later by 50 µl of MDP labelled with $^{99m}Tc$ (100 MBq per ml). Ten rats were killed at 1 and 10 at 90 min after injection and liver and tumour counted as before.

A reference sample of MDP was withdrawn from the stock solution immediately after the hepatic artery injections and counted immediately prior to the samples. The counts were corrected for decay of $^{99m}Tc$ and the results expressed as percentage of the injected dose (% ID) per gram of tissue. Statistical analysis of the distribution of labelled marker between liver and tumour in control and experimental animals was by non-parametric Mann-Whitney test.

### Results

*Mean hepatic replacement*

The mean hepatic replacement by tumour was 27.3 ± 15.2% (mean ± s.d.).

*Angiotensin II*

*Intravenous infusion*

*Distribution of marker* During systemic administration of angiotensin II the percentage injected dose of regionally delivered MDP per gram of liver at one minute (16.0 ± 5.4 × 10−1 mean ± s.d.) was significantly greater ($P < 0.02$) than in control animals (3.55 ± .15. × 10−1). However the percentage injected dose of MDP per gram for tumour in angiotensin treated rats (9.6 ± 3.0 × 10−1) was not significantly greater than that in control animals (5.17 ± 0.55 × 10−1) (Figure 1). The tumour:liver ratio in rats infused with angiotensin II was 0.6:1 compared to 1.5:1 in controls.

*Blood pressure* Arterial blood pressure rose from 117.8 ± 14.8 mmHg to 149 ± 22.4 mmHg after IV infusion of angiotensin II. The time from the commencement of the angiotensin II infusion to the first change in blood pressure was 8.14 ± 7.58 s and the maximal effect was observed at 29.0 ± 19.8 s. In four of the rats, the peak blood pressure was maintained until the animals were killed one minute after the MDP injection.

*Intra-arterial angiotensin II*

*Distribution of marker*

*One minute* The percentage of the injected dose of MDP per gram of normal liver tissue in angiotensin II treated rats (11.1 ± 1.1 × 10−1) was significantly greater ($P < 0.02$) than in control animals (3.55 ± 0.15 × 10−1). Similarly the percentage injected dose of marker per gram in tumour tissue (23.1 ± 5.7 × 10−1) was significantly greater in angiotensin II treated animals ($P < 0.01$) than that in controls (5.17 ± 0.55 × 10−1) (Figure 2). The tumour:liver ratio .at one minute in angiotensin II treated rats was 2.1:1.

*90 minutes* Ninety minutes after the intra-arterial injection of MDP and angiotensin II there was still significant retention ($P < 0.01$) of MDP both in normal liver (6.4 ± 1.4 × 10−1) and in tumour (3.1 ± 1.07 × 10−1) compared to controls normal liver (0.79 ± 0.07 ± 10−1) and tumour (0.61 ± 0.04 × 10−1) (Figure 3).

*Arterial blood pressure* Arterial blood pressure rose from 114.3 ± 14.8 mmHg (mean ± s.d.) to a maximum of 142 ± 22.4 mmHg after the intra-arterial injection of angiotensin II. The time from injection of the IA bolus of angiotensin II to the first change in blood pressure was 8.25 ± 1.47 s and the maximal change in time to peak occurred at 19.25 ± 0.61 s.

**Figure 1** The distribution of marker between liver and tumour after systemic angiotensin II infusion.

**Figure 2** The distribution of marker between liver and tumour after intra-arterial angiotensin injection 1 min after marker injection.

**Figure 3** The effect of intra-arterial angiotensin II on the distribution of marker 90 min after marker injection.

*Intra-arterial phenylephrine*

*Distribution of marker*

*One minute* The percentage injected dose of MDP per gram of marker retained in normal liver in phenylephrine treated rats (9.62 ± 2.5 × 10–1) was significantly greater ($P < 0.02$) than in control animals (3.55 ± 01.04 × 10–1). Similarly, the percentage injected dose per gram of marker retained in tumour (21.7 ± 5.69 × 10–1) was significantly greater ($P < 0.01$) than in control animals (5.17 ± 0.55 × 10–1) (Figure 4). The tumour:liver ratio 1 min after injection of phenylephrine was 2.25:1 indicating relatively greater retention of the marker within tumour than liver.

*90 minutes* Ninety minutes after the bolus injection of MDP there was still significant retention of marker compared to controls both in tumur (6.9 ± 2.6 × 10–1; controls 0.61 ± 0.04 × 10–1) and in normal liver (5.9 ± 0.11; controls 0.79 ± 0.07 × 10–1; $P < 0.01$) (Figure 5).

*Arterial blood pressure*

The mean arterial blood pressure rose from 88 ± 7.4 mmHg (mean ± s.d.) to 135 ± 8.3 mmHg after phenylephrine injection. The time to the first change in blood pressure was 7.83 ± 3.37 s (mean ± s.d.) and the time to maximal response was 14.1 ± 6.48 s.

### Discussion

The prognosis for patients with liver metastases derived from colorectal carcinoma remains poor. Resection of the metastases is rarely feasible and systemic chemotherapy does not in general prolong survival (Hughes *et al.*, 1986; Kemeny *et al.*, 1980), although recent studies of systemic 5-FU and leucovorin have shown a minor survival benefit (Kerr, 1989). Similarly, although regional chemotherapy has produced objective tumour responses in up to 50% of patients it has not be demonstrated to significantly improve survival (Kemeny *et al.*, 1987). A possible explanation of the failure of regional chemotherapy to improve survival in these patients is that there is no preferential delivery of the cytotoxic drug to the tumour (Sigurdson, 1986) since many are relatively hypovascular compared to the surrounding liver parenchyma.

The study by Daly *et al.* which demonstrated improved response rates to regional chemotherapy in patients with hypervascular metastases compared to patients with hypovascular metastases (Daly, 1985) and which reported improved FUDr uptake in metastases which had a high uptake of MAA would appear to support this hypothesis (Daly *et al.*, 1985).

Sasaki infused $^{81}$m Krypton into the hepatic artery of patients with liver tumours and estimated arterial flow to liver and tumour from the detected radioactivity in both tissues (Sasaki *et al.*, 1985). They demonstrated that the ratio of tumour to liver blood flow increased to 3.3:1 after an intra-arterial infusion of angiotensin II. The preferential supply of blood to tumour observed in their study is similar to the results of this study in which angiotensin II and phenylephrine enhanced the retention of an inert marker resembling chemotherapeutic drug by hepatic tumour. Intra-arterial administration of angiotensin II resulted in a significant retention of marker in both normal liver tissue and tumour. One minute after administration the retention of the marker was increased 4-fold within the tumour compared to control animals. Since the retention of marker in tumour

**Figure 4** The distribution of marker between liver and tumour after intra-arterial phenylephrine injection 1 min after marker injection.

**Figure 5** The effect of intra-arterial phenylephrine on the distribution of marker 90 min after marker injection.

was greater than that of normal liver there was also an increase in the tumour: liver ratio to 2.1:1 in experimental animals. Phenylephrine which is also an hepatic arterial vasoconstrictor produced similar results to angiotensin II. One minute after administration of the phenylephrine both tumour and liver retention of marker were increased with a tumour:liver ratio of 2.1:1. The experiments with both agents indicate that regionally delivered vasoconstrictors increase delivery of a marker to liver tumour. The findings of this investigation are similar to our previously reported results with degradable starch microspheres (Cooke & Chang, 1990). Concomitant administration of degradable starch microspheres and marker produced significant retention of marker in both liver and tumour. In this study, 1 min after administration of DSM the tumour:liver ratio was 2.3:1. Angiotensin II, phenylephrine and DSM would therefore appear to effect an intrahepatic redistribution of blood, diverting blood flow and marker towards liver tumour.

Our results are also in agreement with those of Sasaki using intravenous infusions of angiotensin II to manipulate tumour blood flow. Intravenous angiotensin II did not consistently improve the tumour:liver blood flow ratio and was less than 1:1 for the first 2 min of his experiments. The findings are similar to those of this study in which the concentration of marker was greater than in tumour after IV angiotensin II, with a tumour:liver ratio of 0.6:1.

The effects of angiotensin II on systemic and hepatic haemodynamics are transient and of short duration, and the administration of this vasoactive agent is occasionally followed by a rebound hypotension. The maximum change in liver blood flow occurs before the maximum change in blood pressure and the regionally delivered marker must be given during the period when there is maximum alteration in liver blood flow. Ninety minutes after a single IA bolus injection of phenylephrine or angiotensin, although systemic haemodynamics had returned to the baseline values, the retention of marker in both liver and tumour was significantly greater than in control animals. In the control animals, however, the marker was rapidly washed out of both the normal liver and tumour by the hepatic arterial and portal venous flows resulting in minimal retention at 90 min.

This study contrasts with our previously reported findings using DSM where almost all the marker was washed out of the normal liver at 90 min, presumably by restoration of portal venous flow after degradation of the microspheres. It suggests that the effects of arterial vasoconstrictors on the intrahepatic vasculature persist for longer periods than the effects on systemic blood pressure which are transient and only last for approximately 3 min after the injection. Alternatively, the marker may be already outside the vascular space, either in the interstitial tissues or within the cells which results in a reduction of marker wash out. However, this explanation seems unlikely since the marker is washed out of both normal liver tissue and tumour control animals.

The results of this study suggest that hepatic arterial vasoconstrictors can improve drug delivery to liver tumour, especially hypovascular tumours and may improve the results of regional chemotherapy.

This study was supported by the Cancer Research Campaign and North West Cancer Research Fund.

The tumour cell line was a gift from Dr S.A. Eccles, Institute of Cancer Research, Sutton, Surrey, UK, and angiotensin II, a gift from Miss J.A. Goldberg.

### References

ACKERMAN, N.B., LIEN, W.M., KONDI, E.S. & SILVERMAN, N.A. (1969). The blood supply of experimental liver metastases I. The distribution of hepatic artery and portal vein blood to 'small' and 'large' tumours. *Surgery*, **66**, 1067.

BURTON, M.A. & GRAY, B.N. (1987). Redistribution of blood flow in experimental hepatic tumours with noradrenaline and propanolol. *Br. J. Cancer*, **56**, 585.

CHAN, R.C., BABBS, C.F., VETTER, R.J. & LAMAR, C.M. (1984). Abnormal response of tumour vasculature to vasoactive drugs. *JNCI*, **72**, 145.

COOKE, T. & CHANG, D. (1990). Increasing the uptake of a low molecular weight marker in liver tumour by degradable starch microspheres. A possible mechanism of action. *In Progress in Regional Cancer Therapy*, Jakesz, R. & Rainer, M. (eds). Springer-Verlag, pp. 98–104.

ENSMINGER, W.D., ROSOWSKY, A., RASO, V. & 5 others (1978). A clinical pharmacological evaluation of hepatic arterial infusions of 5-fluoro-2-deoxyuridine and 5-fluorouracil. *Cancer Res.*, **38**, 3784.

FINLAY, I.G. & MCARDLE, C.S. (1983). Effect of occult hepatic metastases on survival after curative resection for colorectal carcinoma. *Gastroenterology*, **85**, 596.

HEMINGWAY, D.M., GRIME, S., NOTT, D.M., JENKINS, S.A. & COOKE, T.G. (1989*a*). Hepatic perfusion index: pause for thought. *Br. J. Surg.*, **76**, 1345.

HEMINGWAY, D.M., NOTT, D.M., CHANG, D., JENKINS, S.A. & COOKE, T.G. (1989*b*). The effects of vasopressin on hepatic and tumour haemodynamics in an experimental model of liver tumour. *Br. J. Cancer*, **60**, 467.

HUGHES, K.S., SIMON, R., SONGHORABODI, S. & 46 others (1986). Resection of the liver for colorectal carcinoma metastases: a multi-institutional study of patterns of recurrence. *Surgery*, **100**, 278.

KEMENY, N., DALY, J., REICHMAN, B., GELLER, N., BOTET, J. & ODERMAN, P. (1987). Intrahepatic or systemic infusion of fluorodeoxyuridine in patients with liver metastases from colorectal carcinoma. *Ann. Int. Med.*, **107**, 459.

KEMENY, N., YAGODA, A., BRAUN, D. & GOLBEY, R. (1980). Therapy for metastatic colorectal carcinoma with a combination of Methyl CCNY, 5 fluorouracil, vincristine and streptozotocin (MOF-Strep). *Cancer*, **45**, 876.

KERR, D.J. (1989). 5-Fluorouracil and folinic acid: interesting biochemistry or effective treatment. *Br. J. Cancer*, **60**, 807.

MATTSON, J., APPELGREN, L., HAMBERGER, B., PETERSON, M.I. (1977). Adrenergic innervation of tumour blood vessels. *Cancer Letters*, **3**, 347.

MATTSON, J., APPELGREN, L., KARLSSON, L. & PETERSON, M.I. (1978). Influence of vasoactive drugs and ischaemia on intratumour blood flow distribution. *Europ. J. Cancer*, **14**, 761.

RAPOPORT, A.M. & BURLESON, R.C. (1970). Survival of patients treated with systemic fluorouracil for hepatic metastases. *Surg. Gynecol. Obstetrics*, **130**, 773.

RUSSELL, A.M., TONG, D., DAWSON, L.E. & WISBECK, W. (1984). Adenocarcinoma of the proximal colon. Sites of initial dissemination and patterns of recurrence following surgery alone. *Cancer*, **53**, 360.

SASAKI, Y., INAOKA, S., MASEGAWA, Y. & 5 others (1985). Changes in distribution of hepatic blood flow induced by intra-arterial infusion of angiotensin II in human hepatic cancer. *Cancer*, **55**, 311.

SIGURDSON, E.R., RIDGE, J.A. & DALY, J.M. (1986). Fluorodeoxyuridine uptake by human colorectal hepatic metastases after hepatic artery infusion. *Surgery*, **100**, 285.

TAYLOR, I., BENNETT, R. & SHERRIFFS, S. (1979). The blood supply of colorectal liver metastases. *Br. J. Cancer*, **39**, 749.